# Recombinase Polymerase Amplification Assay for Rapid Diagnostics of Dengue Infection

**DOI:** 10.1371/journal.pone.0129682

**Published:** 2015-06-15

**Authors:** Ahmed Abd El Wahed, Pranav Patel, Oumar Faye, Sasikanya Thaloengsok, Doris Heidenreich, Ponpan Matangkasombut, Khajohnpong Manopwisedjaroen, Anavaj Sakuntabhai, Amadou A. Sall, Frank T. Hufert, Manfred Weidmann

**Affiliations:** 1 Unit of Infection Models, German Primate Center, Goettingen, Germany; 2 Department of Virology, Mansoura University, Dakahlia, Egypt; 3 CBS1-Highly Pathogenic Viruses, Center for Biological Threats and Special Pathogens, Robert Koch Institute, Berlin, Germany; 4 Arbovirus Unit, Pasteur Institute, Dakar, Senegal; 5 Department of Microbiology, Faculty of Science, Mahidol University, Bangkok, Thailand; 6 Department of Virology, University Medical Center, Goettingen, Germany; 7 Systems Biology of Diseases Research Unit at Faculty of Science and Center of Emerging and Neglected Infectious Diseases, Mahidol University, Bangkok, Thailand; 8 Functional Genetics of Infectious Diseases Unit, Institute Pasteur, Paris, France; 9 Institute of Microbiology and Virology, Brandenburg Medical School Theodor-Fontane, Senftenberg, Brandenburg, Germany; 10 Institute of Aquaculture, University of Stirling, Stirling, Scotland, United Kingdom; Naval Research Laboratory, UNITED STATES

## Abstract

**Background:**

Over 2.5 billion people are exposed to the risk of contracting dengue fever (DF). Early diagnosis of DF helps to diminish its burden on public health. Real-time reverse transcription polymerase amplification assays (RT-PCR) are the standard method for molecular detection of the dengue virus (DENV). Real-time RT-PCR analysis is not suitable for on-site screening since mobile devices are large, expensive, and complex. In this study, two RT-recombinase polymerase amplification (RT-RPA) assays were developed to detect DENV1-4.

**Methodology/Principal Findings:**

Using two quantitative RNA molecular standards, the analytical sensitivity of a RT-RPA targeting the 3´non-translated region of DENV1-4 was found to range from 14 (DENV4) to 241 (DENV1-3) RNA molecules detected. The assay was specific and did not cross detect other Flaviviruses. The RT-RPA assay was tested in a mobile laboratory combining magnetic-bead based total nucleic acid extraction and a portable detection device in Kedougou (Senegal) and in Bangkok (Thailand). In Kedougou, the RT-RPA was operated at an ambient temperature of 38°C with auxiliary electricity tapped from a motor vehicle and yielded a clinical sensitivity and specificity of 98% (n=31) and 100% (n=23), respectively. While in the field trial in Bangkok, the clinical sensitivity and specificity were 72% (n=90) and 100%(n=41), respectively.

**Conclusions/Significance:**

During the first 5 days of infection, the developed DENV1-4 RT-RPA assays constitute a suitable accurate and rapid assay for DENV diagnosis. Moreover, the use of a portable fluorescence-reading device broadens its application potential to the point-of-care for outbreak investigations.

## Introduction

Dengue virus (DENV) is a mosquito-transmitted virus that causes mild ((dengue fever (DF)) to severe disease ((dengue hemorrhagic fever (DHF) and dengue shock syndrome (DSS)) in humans [1]. DENV consists of four serotypes (DENV1-4) each comprising multiple genotypes and belongs to the genus *Flavivirus* of the family *Flaviviridae* [2].

Before 1970 DENV outbreaks were reported in nine countries but has since expanded to more than 100 countries, with an estimated 96 million apparent and 294 million unapparent cases in 2010 [3]. Two and a half billion people are at risk of infection worldwide [4]. Since specific treatment or a vaccine are currently not available [5], early detection plays a key role for initiation of control and preventive measures in dengue endemic regions such as mosquito control and social mobilization [6].

DENV is detectable in blood up to 5–7 days after the onsets of symptoms. From the 5^th^ day, diagnosis depends mainly on detection of specific IgM and IgG antibodies using ELISA methods [7, 8]. Therefore, both virus and antibody detection are crucial for the identification of infected cases. The reference method of DENV diagnosis is virus isolation [9, 10], which takes a week and requires highly equipped laboratories. The most widely used methods are antigen detection in ELISA or rapid diagnostic tests (RDTs) formats [8]. Antigen detection is based on the non-structural protein 1 (NS1) of the DENV [11]. RDTs are fast (10–15 minutes) and suitable for point-of-care screening. However, RDTs clinical sensitivity (21–99%) and specificity (77%–98%) vary greatly [7, 8, 12, 13]. The gold standard method for molecular detection of DENV is real-time RT-PCR, which detects DENV RNA within 60–90 minutes [14–16]. Real-time RT-PCR is highly sensitive and specific [14, 16–18]. It requires precautions (carry over prevention systems and physical separation of pipetting sites), sophisticated equipment, and is cold chain dependent, which makes it difficult to implement at the point-of-need.

Isothermal DNA amplification methods represent an alternative to real-time PCR. There is only one development for the application of Nucleic acid sequence based amplification (NASBA) [19] but several applications of reverse transcription Loop mediated isothermal amplification (RT-LAMP) for the detection and differentiation of DENV serotypes have been described [20–23]. RT-LAMP reactions are performed at 60°C, tests run for more than 30 minutes, and results are measured either by turbidity index or by visual qualitative fluorescence detection of SYBR Green [24]. In contrast, real-time recombinase polymerase amplification (RPA) amplifies at 39–42°C and uses a fluorescent exo-probe for detection [25]. The RPA assay is very fast (3–15 minutes) and can be operated on a portable device, the tubescanner (19x17.5 cm). In this study, a-point-of-need reverse transcription RPA (RT-RPA) assays for the detection of DENV1-4 without differentiation between the serotypes were developed and evaluated with samples from Senegal and Thailand. A mobile RPA unit was deployed to Kedougou in Senegal and to Bangkok, Thailand.

## Methods

### Ethics Statement

Human samples tested in Senegal were provided by the WHO collaborating center for Abovirus and Viral Hemorrhagic Fever at the Institute Pasteur de Dakar (IPD). The IPD has the required ethical approvals from the Senegalese National Health Research Council and the signed consent forms from patients. The ethical committee refernce number is 2472. Human samples tested during the field trial in Thailand were used after an approval of Faculty of Medicine, Vajira Hospital, Bangkok, Thailand Research Ethics Committee (Certificate of approval number: COA29/2012) and signed consent forms from patients.

### Viruses

Robert Koch Institute, Berlin, Germany provided the viruses and/or nucleic acids used in this study. Viruses are listed in Table 1.

**Table 1:** List of viral RNA tested.

Name	Strain	DENV1-3 RT-RPA	DENV4 RT-RPA
Dengue virus serotype 1	VR344 (Thai 1958)	+	-
Dengue virus serotype 2	VR345 (TH-36)	+	+
Dengue virus serotype 3	VR216 (H87)	+	-
Dengue virus serotype 4	VR217 (H241)	-	+
Yellow Fever virus	17D	-	-
Yellow Fever virus	Asibi	-	-
Tick Borne Encepahlitis virus-FE	Far eastern subtype	-	-
Chikungunya virus	LR 2006	-	-
Zika virus	MR766	-	-
Tick-borne encephalitis virus	K23	-	-
Japanese encephalitis virus	ATCC SA14142	-	-
West Nile virus	Israel	-	-

DENV1-3 RT-RPA assay detected DENV1-3 but not other viral RNA. DENV4 RT-RPA identified DENV2 and DENV4.

+, positive;-, negative.

### Generation of molecular RNA standard

According to a previously published protocol, two *in vitro* transcribed RNA standards were prepared [26, 27]. PCR primers used for the amplification of the target region for ligation into the TA cloning vector pCRII are listed in Table 2. DENV1-3 and DEN4 RNA molecular standards cover 242 and 248 bp of the 3’ non-translated region (3’NTR) (10462–10703 nt and 10398–10645 nt of GenBank accession numbers AY662691.1 and GU289913.1), respectively. The RNA standards were tested by using a published real-time RT-PCR [28]. The Light Cycler 2.0 and the LightCycler 480 RNA Master Hydrolysis Probes kit (Roche, Manheim, Germany) were used. The following temperature profile was used: RT step 63°C/3 minutes, initial activation at 95°C/30 seconds, 45 cycles of 95°C/15 seconds and 60°C/60 seconds, and a final cooling step of 40°C/30 seconds.

**Table 2:** Sequence of primers and probes for the construction of molecular standards, real-time RT-PCR and RT-RPA assays.

Name	Sequence 5’-3’	Amplicon size bp
DENV1-3-STD-UP	CTGTACGCACGGTGTAGCAGAC	242
DENV1-3-STD-DP	CCTGTTGATTCAACAGCACCATTC	
DENV4-STD-UP	CTGTACGCGTGGCATATTG	248
DENV4-STD-DP	CCTGTTGGATCAACAACACC	
DENV-PCR-FP	AAGGACTAGAGGTTAKAGGAGACCC	85
DENV-PCR-RP	CTGHRGAGACAGCAGGATCTCTG	
DENV-PCR-P	FAM-AACAGCATATTGACGCTGGGARAGAC-TAMRA	
DENV1-3-RPA-FP13	ATTCAACAGCACCATTCCATTTTCTGGCGTTCTGTG	97
DENV1-3-RPA-RP4	AACAGCATATTGACGCTGGGAGAGACCAGAGATC	
DENV4-RPA-FP3	CATCTTGCGGCGCTCTGTGCCTGGATTGA	86
DENV4-RPA-RP2	CACAAAAACAGCATATTGACGCTGGGAAAG	
DENV-RPA-P3	ATATTGACGCTGGGAGAGACCAGAGATCCTGC(BHQ1-dT)(THF)(FAM-dT)CTCCTCAGCATCATTC-(Phosphate)	N/A

Bp, base pair; DENV1-3-STD-UP/DP are for DENV1-3 RT-PCR; DENV4-STD-UP/DP are for DENV4 RT-PCR; DENV-PCR-FP/RP/P, real-time RT-PCR primers and Taqman probe (FAM/TAMRA); DENV1-3-RPA-FP, forward and reverse primers for DENV1-3 RT-RPA; DENV4-RPA-FP, forward and reverse primers for DENV4 RT-RPA; DENV-RPA-P, exo-probe; BHQ1-dT: thymidine nucleotide carrying Blackhole quencher1, THF: tetrahydrofuran spacer, FAM-dT: thymidine nucleotide carrying Fluorescein. N/A, non applicable.

### RT-RPA primers and exo-probes

Two RT-RPA assays were developed to cover DENV1-4, one to detect DENV1-3 and another to detect DENV4. The assays were not used for distinguishing between DENV serotypes. Nineteen forward primers (FP), 5 reverse primers (RP), and 3 exo-probes (exo-Ps) (S1 Fig) were used to select the combination producing the highest analytical sensitivity for the DENV1-3 RT-RPA assay. Three FP, 2 RP, and 2 exo-P were tested for the DENV4 RT-RPA assay (S2 Fig). Oligonucleotides were synthesized by TIB MOLBIOL (Berlin, Germany).

### RT-RPA assay conditions

In the laboratory, the RT-RPA assay was carried out using the TwistAmp exo kit (TwistDx, Cambridge, UK) and the reverse transcriptase (RT) Transcriptor (Roche, Mannheim, Germany) was added as described [29, 30]. In the field trials in Senegal and Thailand, the RT-RPA assay was performed using the ready-to-use TwistAmp RT exo (TwistDx, Cambridge, UK) as described previously [26].

### Analytical sensitivity of RT-RPA assays

A dilution range from 10^7^ to 10^1^ RNA molecules/μl of the molecular standards in eight replicates was used to determine the analytical sensitivity of RT-RPA assays. A semi-log and probit regression analyses were performed as stated in the statistical analysis section (see below).

### Specificity and cross-reactivity of RT-RPA assays

The specificity of the DENV RT-RPA assays was determined by testing 64 DENV-real-time RT-PCR negative plasma samples (41 samples during the field trial in Thailand and 23 in Senegal). In addition, genomic RNA of the Yellow Fever virus, Tick-borne encephalitis virus, Tick-borne encephalitis virus-Far East, Chikungunya virus, Zika virus, Japanese encephalitis virus and West Nile virus were tested for cross-reactivity.

### Differentiation between specific and non-specific signals of the RT-RPA

**Fig 1:**
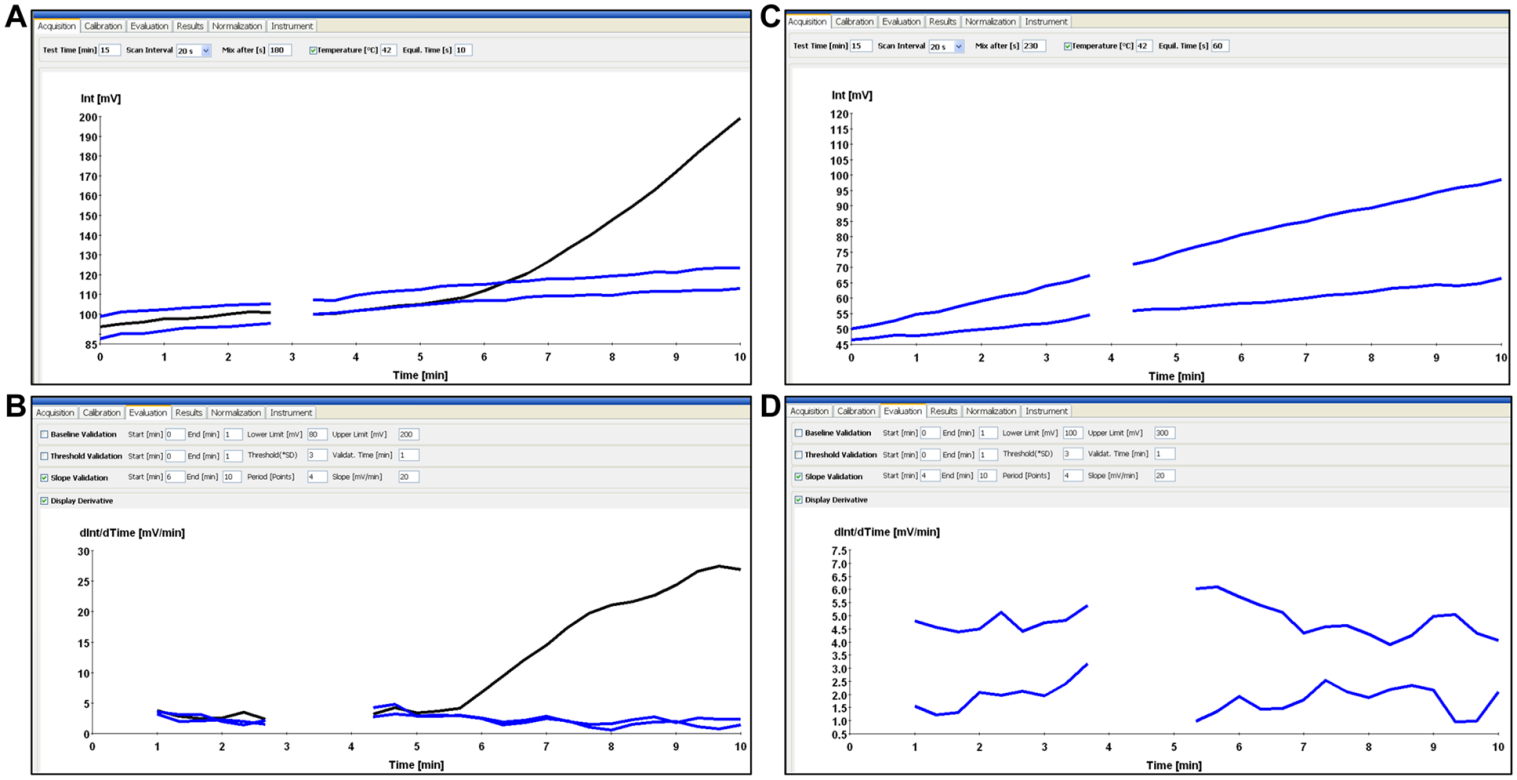
Differentiation between specific and non-specific signals of the RT-RPA assay. A and C are real-time fluorescence intensity; B and D are the 1^st^ derivative analysis. Specific DNA amplification represented by progressive fluorescence development in both views, A and B, while non-specific not. Black line shows specific amplification where blue line shows no amplification.

Real-time fluorescence development in the RT-RPA reaction was measured with the tubescanner (Qiagen Lake Constance GmbH, Stockach, Germany). Fluorescence signals were analyzed by using the tubescanner studio software (Qiagen Lake Constance GmbH, Stockach, Germany). It allows an analysis of accumulative fluorescence intensity over time by threshold validation to identify the increase of fluorescence over time above the mean background signal (raw data, Fig 1A and 1C). Additionally, slope validation is used to verify that the increase of fluorescence occurs at a sufficiently high rate, this can be displayed as a 1^st^ derivative analysis (1^st^ derivative analysis, Fig 1B and 1D).

Specific amplification (black curves Fig 1) should present ascending curves indicating increase of florescence over time in both analyses. Negative or non-specific amplification (blue curves Fig 1) might in a few cases develop fluorescence in the raw data view but never in the 1^st^ derivative analysis. The derivative analysis is therefore necessary to confirm true amplification.

### RT-RPA mobile laboratory

**Fig 2:**
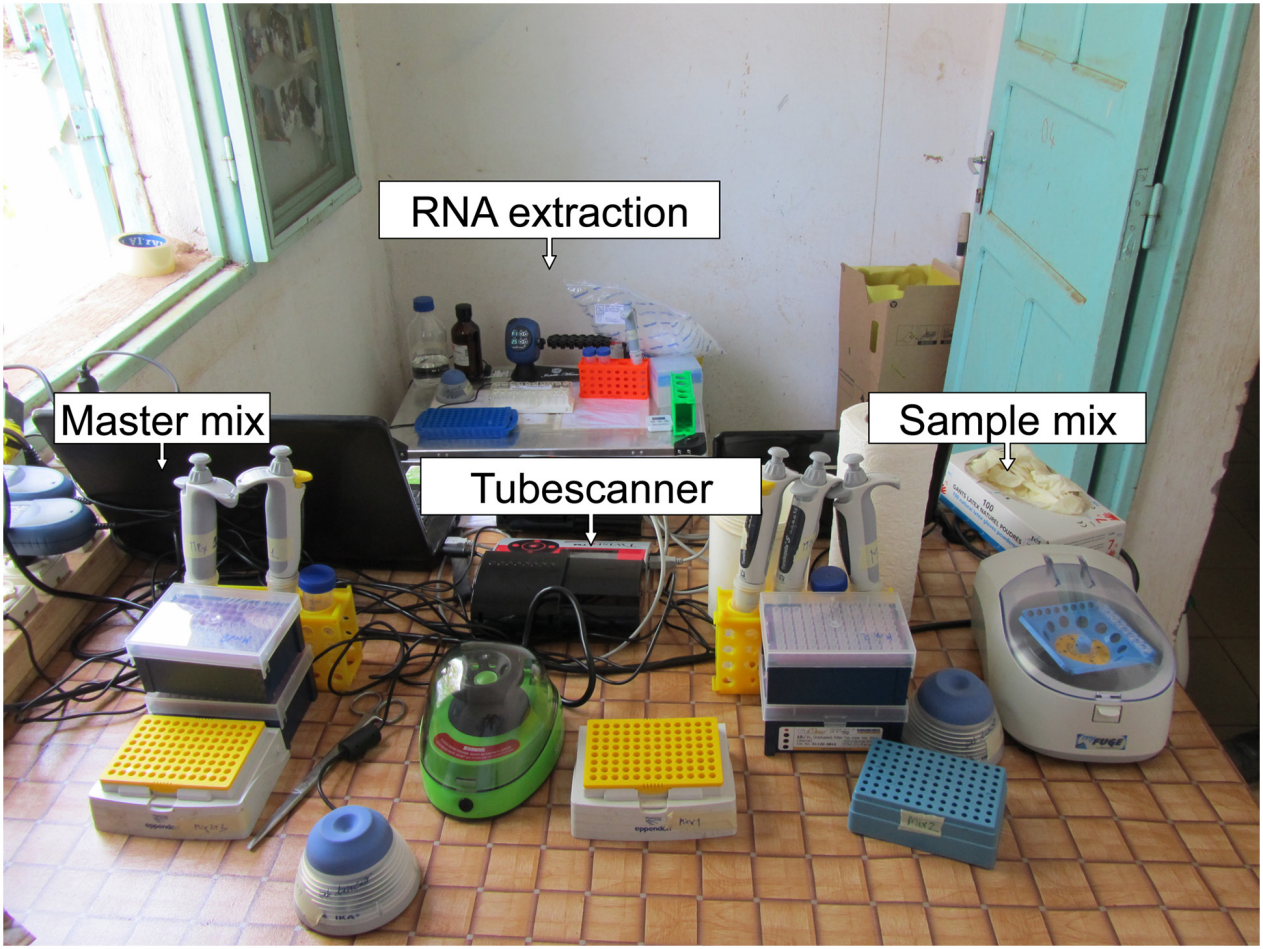
RPA mobile laboratory. The extraction area encompassing magnetic separator stand, vortex, rotator, 1.5–2 ml eppendorf tube rack, automatic 100–1000 μl micopipette, micropipette tips, digital timer, 1.5 ml disposable plastic Eppendorf tubes, and a waste container with autoclavable plastic bags. Both master mix and sample mix areas contain vortex, minicentrifuge, automatic 1–10 and 10–100 μl micopipettes, micropipette tips, scissor, and 0.2 ml tubes rack. The detection was done using the tubescanner (Twista device, TwistDx, Cambridge, UK). In addition to a waste container, gloves, disposable towels, and aluminum box with trolley (740x490x450 mm, ZARGES, Weilheim, Germany).

The mobile laboratory was organized into four sites in close proximity, which included the extraction, the master mix, the sample mix, and the detection-sites (Fig 2). The RNA extraction was done by using a extraction kit based on magnetic beads (Dynabeads SILANE viral NA, Life Technologies, Darmstadt, Germany) according to the manufacturer instructions. The DENV RT-RPA assays were performed by using the ready-to-use TwistAmp exo RT kits (TwistDx, Cambridge, UK), dried DENV1-3 and DENV4 *in vitro* transcribed RNA (using the RNAstable kit, Biomatrica, Inc. San Diego, CA, USA), and dried RT-RPA oligonucleotides (TIB MOLBIOL, Syntheselabor, Berlin, Germany).

Oligonucleotide sets were ordered as dried pellets at an absolute concentration of 840 pmol primers/400 pmol probe. The addition of 200 μl water yielded a stock solution of 4.2 μM/2 μM from which the final concentration of 420 nM/200 nM of the RT-RPA reaction was derived by 10-fold dilution. One 200 μl 40x stock solution thus allowed pipette eight 9-volume master mixes to fill eight 8-tube strips for the tubescanner device. For the positive control, one volume of the master-mix was added to a dried positive control tube.

All reagents used for the mobile laboratory were cold-chain independent, i.e. used stored, and transported at ambient temperature. Electricity was either tapped from a motor vehicle battery via inverter (HPL 1200-D-12 inverter 12V 1200W) or supplied via solar panel and power pack (Yeti 1250 set, GOALZERO, South Bluffdale, UT, USA).

### Field trial in the Kedougou region, Senegal

The mobile RPA was tested in the field station of the IPD in Kedougou, Senegal. Additionally, the mobile RPA laboratory was transferred to a health care station in Bandafasi, Kedougou for use in very simple conditions with auxiliary electricity tapped from a motor vehicle. Inactivated DENV1-4 cell culture supernatants (heating at 56°C/1 hour and gamma irradiation with 30 kGy) and thirty-one DENV RNA positive samples were tested in triplicate.

### Evaluation of RT-RPA assay using RNA extracts in Bangkok, Thailand

RNA extracted from ninety selected samples, positive by SD Dengue Duo Rapid Test (Standard Diagnostic, Inc, Republic of Korea), by QIAamp Viral RNA Mini Kit (Qiagen, Hilden, Germany) and stored between 6–18 months at -80°C. RNA was retested simultaneously with real-time RT-PCR and the RT-RPA assays, each sample was tested at least twice. The real-time RT-PCR was performed by using the Light Cycler 480 RNA Master Hydrolysis Probes kit (Roche, Germany) using the following temperature profile: 61°C for 10 minutes, 95°C for 2 minutes followed by 45 cycles of 95°C for 15 seconds and 60°C for 30 seconds on a 7500 Fast Real-Time PCR system. Data were analyzed using the 7500 Fast Real-Time PCR software provided by Applied Biosystem 7500 Fast Real-Time PCR system (Life technologies, USA). The primers and probe targeted the NS5 gene (S1 Table, [14]).

### Statistical analysis

The semi-log regression analyses of the analytical sensitivity of RT-RPA assays were performed using PRISM (Graphpad Software Inc., San Diego, CA, USA) and the probit anylsis by STATISTICA (StatSoft, Hamburg, Germany). Comparison between real-time RT-PCR and RT-RPA for the detection of DENV was performed by linear regression analysis using Prism.

## Results

### Analytical sensitivity of DENV1-3, and DENV4 RT-RPA assays

**Fig 3:**
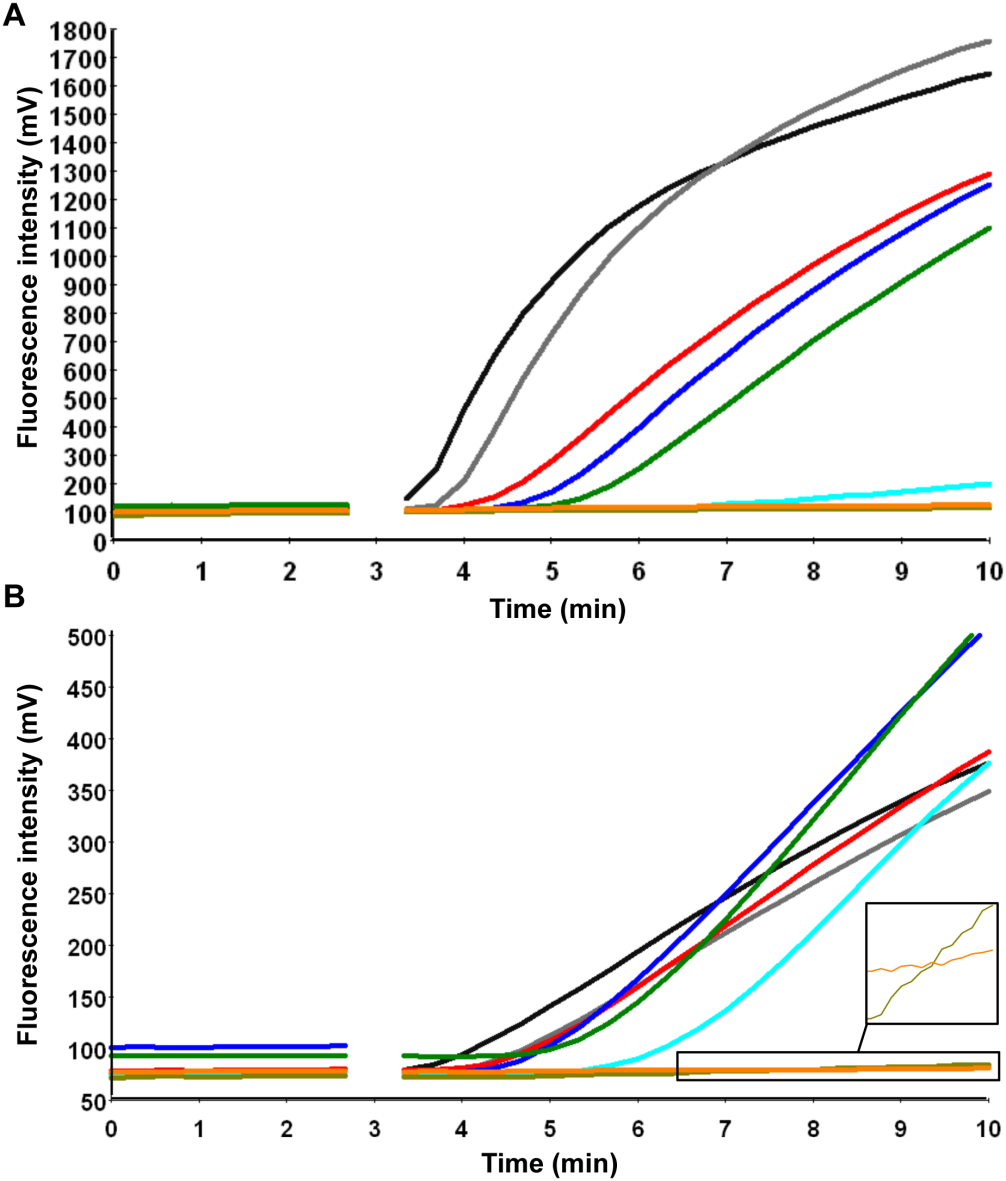
Analytical sensitivity of DENV RT-RPA assays. A, DENV1-3 and B, DENV4 RT-RPA assays. Fluorescence development via real-time detection in one RT-RPA run by using a dilution range of 10^7^–10^1^ RNA molecules/μl of the DENV1-3 and DENV4 RNA molecular standards (Graph generated by ESEquant tubescanner studio software). The sensitivity was 100 and 10 RNA copies for DENV1-3 and DENV4 RT-RPA, respectively. Data of 8 RT-RPA runs is compiled in Fig 4. The signal for ten RNA copies is very weak. The box in the lower right corner of Fig 3B magnifies the fluorescence signals for the ten RNA copies and the negative control. 10^7^ represented by black line; 10^6^, gray; 10^5^, red; 10^4^, blue; 10^3^, green; 10^2^, cyan; 10^1^, dark khaki; negative control, orange.

**Fig 4:**
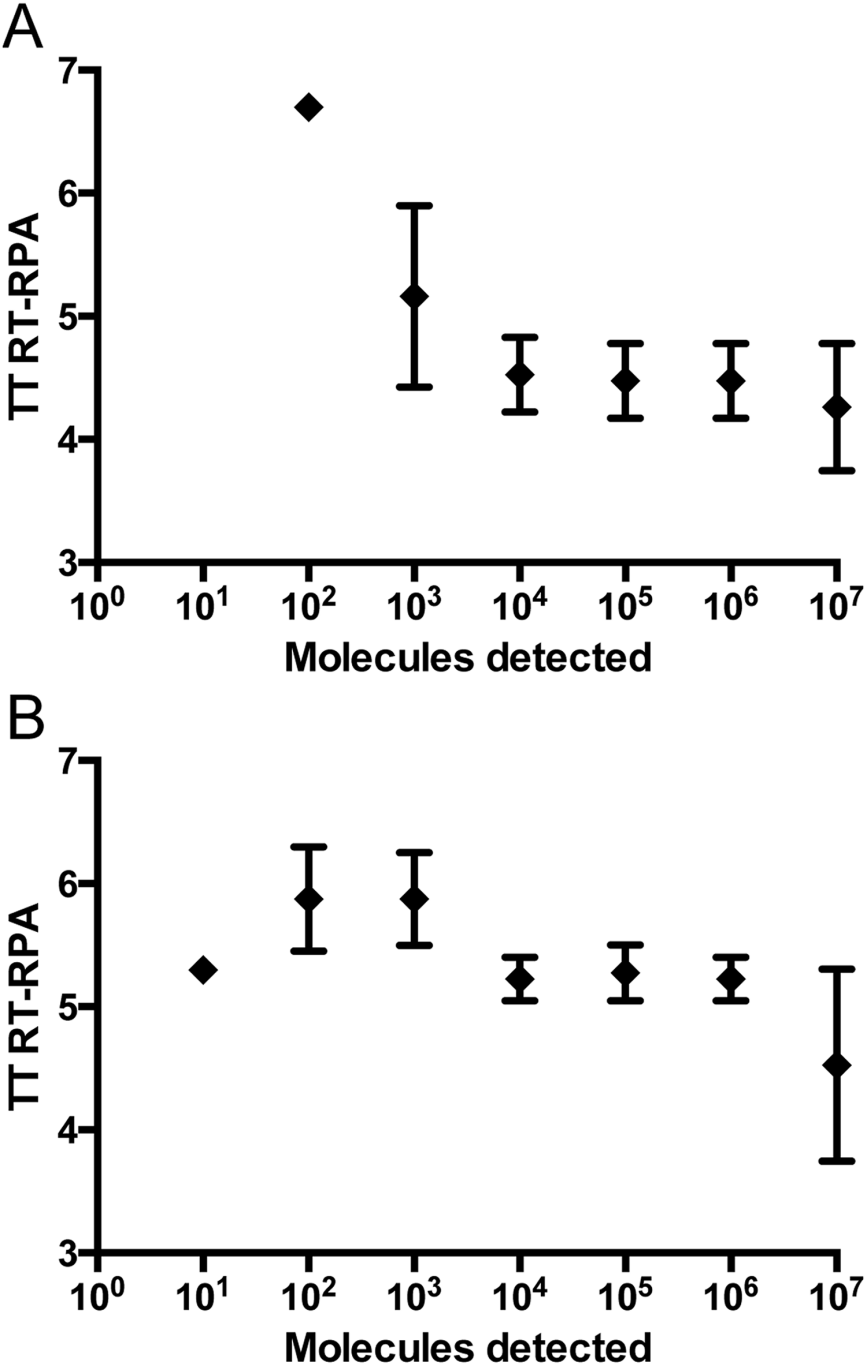
Reproducibility of DENV RT-RPA assays. A, DENV1-3 and B, DENV4 RT-RPA assays. Semi-logarithmic regression of the data collected from eight DENV RT-RPA test runs on the RNA standard using PRISM. Both assays yielded results between 3–7 minutes. In DENV1-3 RT-RPA assay, 10^7^–10^3^ RNA molecules were detected 8 out of 8 runs, 10^2^ in 1 out of 8 and 10 copies was not identified. In DENV4 RT-RPA assay, 10^7^–10^2^ RNA molecules were detected 8 out of 8 runs and 10 copies in 6/ out of 8. In Fig 4B, the value for 10 RNA copies was consistently 5.3 minutes in all 6 cases.

**Fig 5:**
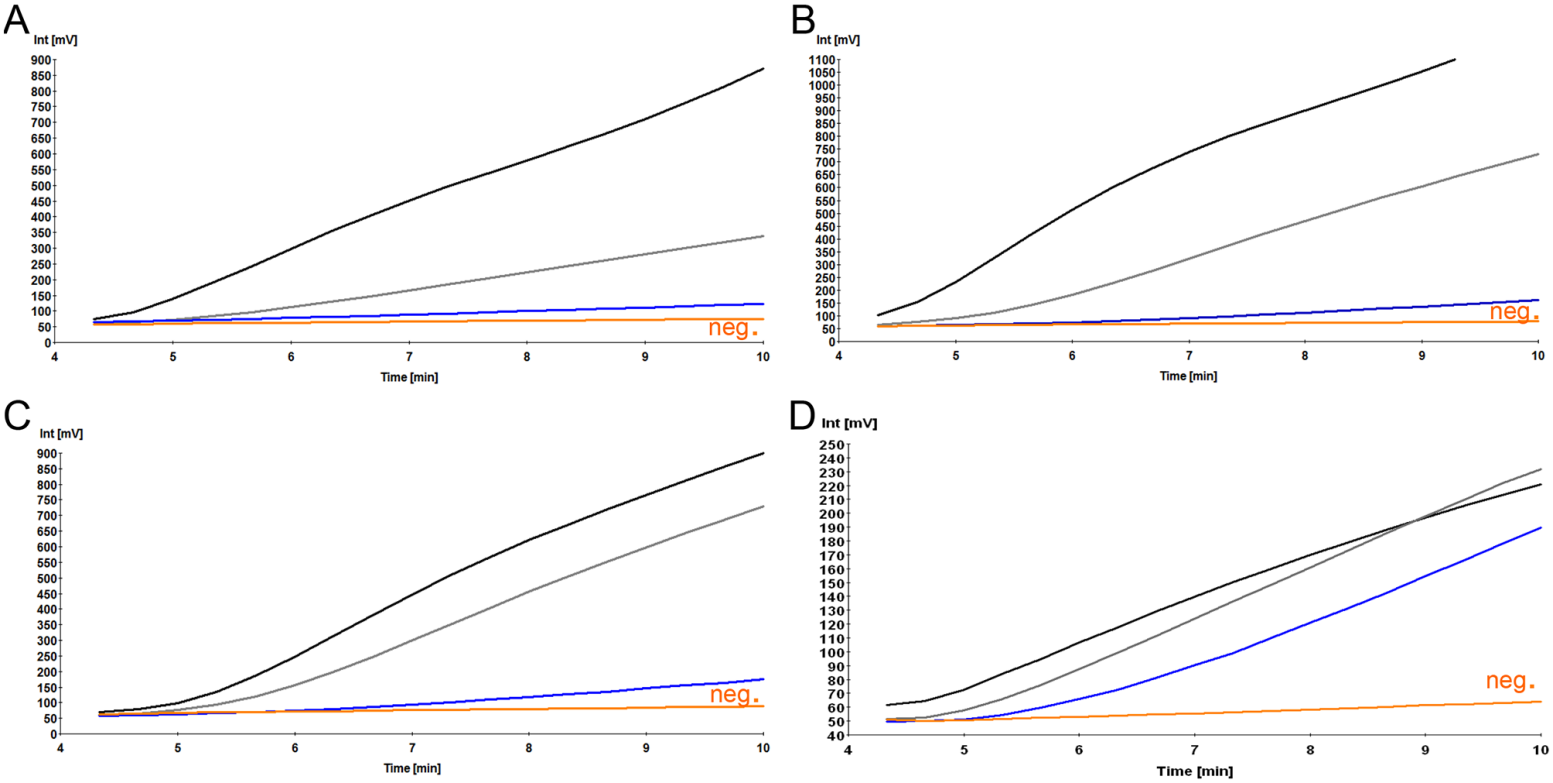
Performance of DENV RT-RPA assays on spiked plasma samples. A, sample spiked with DENV1; B, DENV2; C, DENV3; D, DENV4. DENV serotypes 1–4 were spiked into plasma samples. Serial dilutions of each of the spiked sample were tested simultaneously with real-time RT-PCR and RT-RPA assays (S2 Table). Limits of detection in RT-RPA assays were 237, 618, 363, and 383 RNA copies of DENV serotypes 1, 2, 3, and 4, respectively.

Due to the variability of DENV sequences in the 3’NTR, two RT-RPA assays were designed, one for the detection of DENV1-3 and one for the detection of DENV4. The primers DENV1-3-RPA-FP13 and RP4 and the primers DENV4-RPA-FP3 and RP2 both in combination with DENV-RPA-P3 yielded analytical sensitivities between 10–100 RNA molecules (Table 2 and Fig 3). Most of the other primer and exo-P combinations produced either non-specific amplification (S3 Fig) or analytical sensitivity between 10^5^–10^3^ RNA molecules (S4 Fig). Using the data of eight RT-RPA runs on the quantitative RNA standard, a semi-log regression (Fig 4) and probit regression analysis were performed. For both assays, the runtime was 3–7 minutes at an efficiency of 0.24 and 0.27 calculated from the linear slopes (-0.2366 and -0.1655) of the semi-log standard regression (E = 10^(-1/slope)-^1) for DEN1-3 and DEN4 RT-RPA assays, respectively. Probit regression yielded a sensitivity at 95% of 241 and 14 RNA molecules detected for the DENV1-3 and the DENV4 RT-RPA assays, respectively. Additionally, inactivated whole DENV1-4 (strains are listed in Table 1) were spiked into plasma samples. Serial dilutions of each spiked sample were tested with real-time RT-PCR and RT-RPA assays simultaneously (S2 Table). Limits of detection in RT-RPA were 237, 618, 363, and 383 RNA molecules detected/reaction of DENV serotypes 1, 2, 3, and 4, respectively (Fig 5).

### Specificity and cross-reactivity of DENV1-3, and DENV4 RT-RPA assays

Sixty-four DENV-PCR negative samples were tested by both DENV RT-RPA assays. Non-specific amplification was not detected. DENV1-3 RT-RPA assay detected DENV serotypes 1, 2, and 3, while DENV4 RT-RPA identified DENV serotypes 2 and 4 (Table 1). No cross detection with viruses listed in Table 2 was observed.

### RPA mobile laboratory in Kedougou region in Senegal

In the field, setting up the mobile laboratory including hooking electricity from a motor vehicle battery took about 20 minutes. A magnetic beads based extraction method was used to avoid the use of a centrifuge and the generation of aerosols.

**Fig 6:**
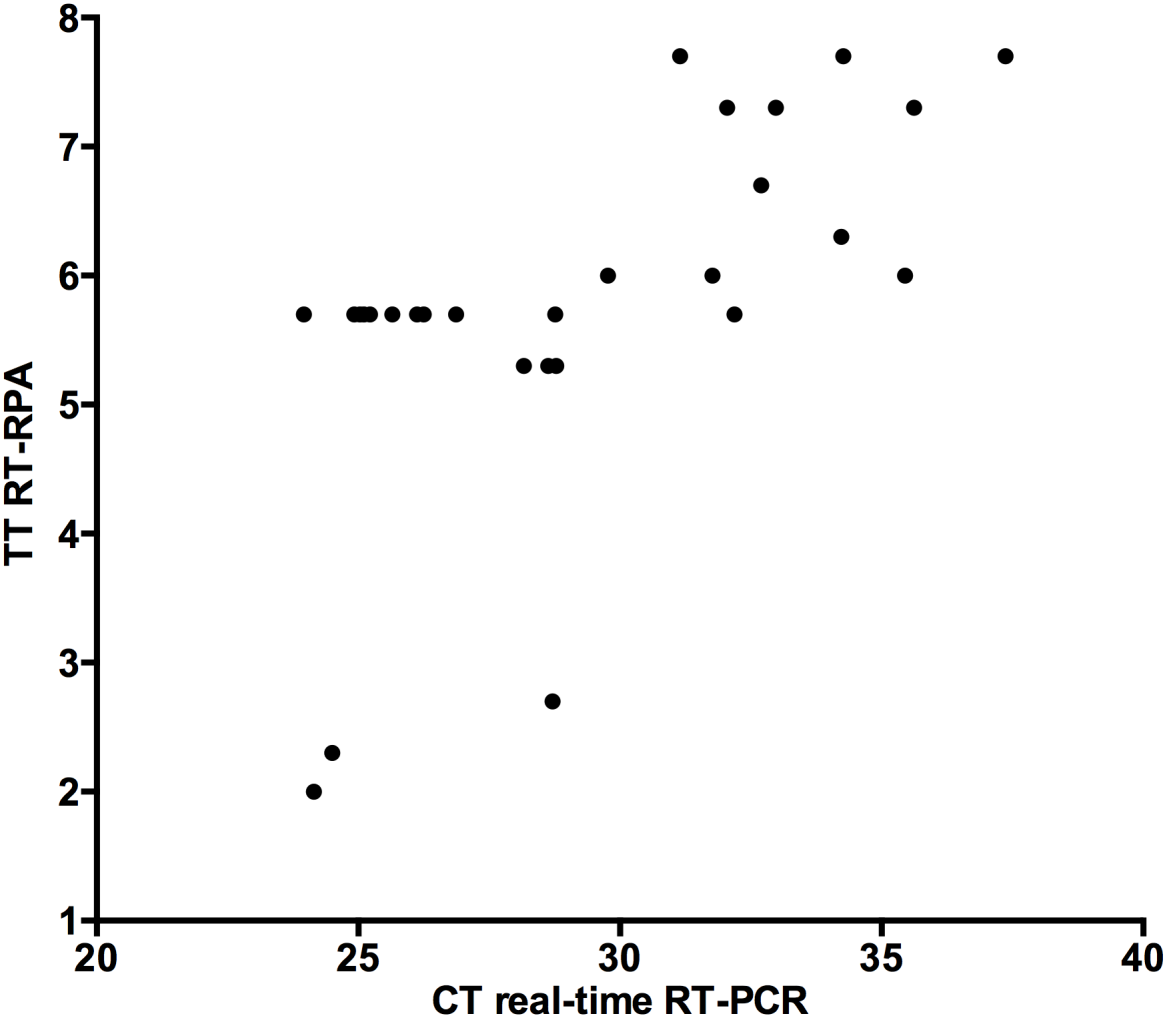
Comparison between real-time RT-PCR (X-axis) and RT-RPA (Y-axis) for the detection of DENV in 31 clinical samples in Senegal. Linear regression analysis of real-time RT-PCR cycle threshold values (Ct, X-axis) and RT-RPA threshold time in minutes (TT, Y-axis) were determined by PRISM (R^2^ = 0.39). The RT-RPA is much faster than the real-time RT-PCR even with samples with high Ct value.

Using the inactivated DENV1-4 spiked plasma as well as 31 DENV positive samples, the DENV RT-RPA assays and the mobile laboratory format were tested in a healthcare center without electricity in Bandafassi (S5 Fig) to simulate an outbreak situation. Extraction and RT-RPA assays were carried out at an ambient temperature of 38°C. The RT-RPA assays were used successfully to detect DENV RNA in the spiked plasma. In comparison to the real-time RT-PCR results, 30 out of 31 DENV-3-positive samples were positive in RT-RPA assays (clinical sensitivity 98%, Fig 6). Samples with high cycle threshold (Ct) value (35–38) in real-time RT-PCR were detected by RT-RPA in a maximum of 8 minutes.

### Assay performance on RNA extracts in Thailand

In Bangkok, Thailand, the RNA of ninety plasma samples extracted and tested between 2012–2013 by RT-PCR, and stored at -80°C was tested. RNA was simultaneously screened by RT-RPA assays and serotype specific real-time RT-PCR (S1 Table). The sensitivity was 72% and 94.4% for RT-RPA and real-time RT-PCR, respectively (S3 Table). RT-RPA assays sensitivities for DENV serotypes were 66.6% (16/24), 44% (4/9), 80.9% (34/42), and 60% (6/10) for DENV1, 2, 3, and 4, respectively. Five samples were only positive in RT-RPA and therefore the DENV serotype was not determined. No correlation was found between threshold time values of RT-RPA and Ct of the real-time RT-PCR (S6 Fig, R^2^ = 0.179).

## Discussion

In their review, Peeling et al., [8] mention characteristics of the future diagnostic test for early detection of DENV. It should be rapid, sensitive, specific, stable at high temperatures (>30°C), and cheap. Currently point-of-care DENV detection is carried out with RDT assays. RDTs are simple and rapid but of limited sensitivity and specificity [7, 13]. Real-time RT-PCR is highly sensitive, usually performed in highly equipped laboratories and not suitable for point-of-care testing. In contrast, RPA technology is as sensitive and specific as real-time RT-PCR, isothermal and easy to use at point-of-need [26, 29–35]. In addition, RPA is fast (3–5 minutes including the reverse transcription step) and can be performed on a portable device (tubescanner). In this study, two RT-RPA assays for detection of DENV (DENV1-3 and DENV4) were developed which cover but do not differentially determine DENV1-4, as required for molecular epidemiological studies. Distinguishing DENV1-4 at the point-of-care is not clinically relevant. The assays were very sensitive with a respective limit of detection of 241 and 14 molecules detected, highly specific, and were evaluated as mobile tests in a low resource setting in Kedougou in Senegal.

Several isothermal amplification technologies were developed in the last two decades, e.g. RT-LAMP [24], NASBA [36], nicking enzyme amplification reaction [37], rolling circle amplification methods [38], helicase dependent amplification [39], and RPA [25]. They differ in the amplification temperature, run time, number of primers used for DNA amplification, detection method (probe-based or intercalating dye dependent) and commercial availability.

Many RT-LAMP assays have been developed for DENV detection using between 6–24 primers [20–23] and yielding results in 30 minutes based on visual inspection (turbidity index, fluorescence) [20–23]. While SYBR Green detection of the RT-LAMP reaction could basically also be done with the tubescanner, RPA is much faster (3–15 minutes) and utilizes only three oligonucleotides (two primers, one exo-P). Recent advances in software development to assist LAMP design may help overcome challenges for highly variant viruses, the tolerance for mismatches in LAMP assays however still needs to be explored [40]. The tolerance of RPA oligonucleotide sets and in particular, of the exo-P probe portions to mismatches [26, 33] are advantageous for RPA amplicon design but remain subject to testing.

Previous work had shown that for RNA viruses the best sensitivity is achieved with an exo-P, encompassing 15 nt 5’ (short section), and 30 nt 3’ (long section) of a basic site mimic (Tetrahydrofuran, THF) and a phosphate at the 3’ end of the exo-P [25, 26]. The phosphate blocks any primer type interference of the long section with the RPA reaction after Exonuclease nicking [25]. In contrast of all tested probes, DENV-RPA-P3 consisting of the standard inverse exo-P structure with a long 32 nt section 5’ and a short 16 nt section 3’ of the THF site (Table 2) yielded the highest sensitivity. Here the long section can act as a primer and contribute to, the sensitivity of the assay [25] as observed for other RPA assays [29, 30].

Of all reverse primers tested DENV1-3 RP4 and DENV4 RP2 yielded the highest assay sensitivities although overlapping the long exo-P 3’ section of DENV-RPA-P3 by 28 nt (S1 and S2 Figs). The best forward primer for the DENV4 assay DENV4 FP3 overlapped the short exo-P 3’ end by 6 nt (S2 Fig). Non-specific amplification was not observed in any of these cases. Therefore, the selection of primer and probe format is still down to trial and error. The factors that determine efficient strand invasion may be a combination of binding enthalpy and local secondary structure. Although advances in calculating these values for PCR primers have been made, they cannot as yet be deduced for RPA [41].

DEN RPA P3 is shared by both assays but carries 4 mismatches (S2 and S7 Figs) in the smaller 13 nt 3’ section of the probe. Efficient RPA tolerates mismatches per primer which should not accumulate at the termini or at the center of the primers and in concordance with what is known about PCR primers mismatches at the 3’ end of the primers impede the RPA reaction [42]. As a whole RPA amplification systems appear to tolerate the presence of 5–9 mismatches in a primer and probe set [26, 33]. We assume that the long 32 nt 5’ section of DEN RPA P3, which matches almost perfectly to DENV1-4, allows for tolerance of the accumulation of the mismatches in the smaller 3’ portion of the probe since it does not conflict with the principle of dissociation of the smaller section the exo-P after Exonuclease action. Shifting the mismatches into the smaller section of the probes may thus be exploited for the design of exo-P for the detection of other highly variable RNA target sequences. In contrast, real-time PCR, which utilized shorter primers and probes, is more sensitive to the presence of mismatches in the target sequence [43]. The linearity of the assay also does not represent a correlation of linearity and sensitivity as seen in real-time PCR assays [29].

Real-time RT-PCR can be used for both qualitative and quantitative analysis of RNA in a sample. In contrast, RT-RPA can currently only be used for qualitative detection of RNA. As shown in Fig 6, the TT values of the RT-RPA can be classified into two groups, before 3 and after 5 minutes. The main reason is the interruption of the fluorescence read by the mixing step after three minutes, which is crucial for the assay sensitivity. During mixing, fluoresces signal are not measured (Figs 3 and 6), therefore, TT values can`t be calculated. This however does not lead to false negatives.

The characteristics of the mobile laboratory (23kg including the aluminium case) are 1) easy transportation by car and airplane, 2) power source from motor vehicle batteries or solar panel with power pack, 3) easy implementation in low resource settings. A magnetic bead extraction was applied to avoid the creation of aerosols and because trials at the Institute Pasteur in Paris in a climate chamber have shown that most commercial centrifuges short-circuited at 80% humidity and temperatures above 38°C (Jean-Claude Manuguerra, personal communication). All reagents were cold chain independent and performed well at 38–40°C ambient temperature. We were able to run the laboratory in the open air and observed no influence of dust on the assay quality. We will continue to follow this concept to allow easy deployment of up to date molecular detection to infrastructure poor settings. Further work will attempt to reduce pipetting steps by drying RPA primers and probe into the RPA reagent pellet, implementing a multiplex RT-RPA assay combining the two DENV RPA assays into one reaction and identifying even simpler extraction protocols.

The DENV RT-RPA assays were evaluated with plasma samples and RNA extracts in Senegal and Thailand. The RT-RPA clinical sensitivities were 98% for samples tested in Senegal and 72% for RNA extracts in Thailand, whereas real-time RT-PCR sensitivity was 98% and 94.4%, respectively. The lower clinical sensitivity of the RT-RPA assays on the RNA extracts in Thailand might not be due to the assay detection limit, which is between 10–100 RNA copies (Figs 3, 4 and 5). As shown in S3 Table, most of the samples, which were negative in RT-RPA had Ct values between 14–30 in real-time RT-PCR. Genetic variations between Asian and African DENV strain could not be the reason for the difference in the assay sensitivity [44–47]. As mentioned above, the RPA targeted the conserved 3’ NTR of the DENV genome and RPA amplification can tolerate 5–9 mismatches. In Senegal, RNA was extracted from plasma samples and screened directly with the RT-RPA assays, while in Thailand, RNA stored at -80°C for 6–18 months was tested. The integrity of the RNA is affected by freezing and thawing, albeit, freezing of clinical samples has a less pronounced effect [48, 49]. It appears that in contrast to real-time RT-PCR [50], RT-RPA performance is influenced by the quality of the RNA confirming an observation we described earlier [26].

In conclusion, two DENV RT-RPA assays were developed for rapid identification of DENV1-4. RPA was easy to implement in low resource settings and high ambient temperatures did not affect its performance.

## Supporting Information

S1 Fig DENV1-3 RT-RPA primers and probes sequence aligned with the DENV1-3 amplicon. Three RPA exo probes (P), 19 forward primers (FP), and 5 reverse primers (RP) were tested to select combinations yielding the highest analytical DENV1-3 RT-RPA sensitivity. FP13, RP4, and P3 produced the best RT-RPA assay sensitivity. NNN are sites of the quencher and fluorophore in following order (BHQ1-dT) (Tetrahydrofuran) (FAM-dT). RC is the reverse complementary of the original sequence used in the experiment.
(DOCX)

S2 Fig DENV4 RT-RPA primers and probes sequences aligned with the DENV4 amplicon. Two RPA exo probes (P), 3 forward primers (FP), and 2 reverse primers (RP) were tested to select combinations yielding the highest analytical DENV4 RT-RPA sensitivity. FP3, RP2, and P3 produced the best RT-RPA assay sensitivity. NNN are sites of the quencher and fluorophore in following order (BHQ1-dT) (Tetrahydrofuran) (FAM-dT). RC is the reverse complementary of the original sequence used in the experiment.
(DOCX)

S3 Fig DENV1-3 RT-RPA assay with non-specific amplification. Fluorescence development via real-time detection by using a dilution range of 10^7^–10^1^ RNA molecules/μl of the DENV1-3 molecular standard. 10^7^ represented by black line; 10^6^, gray; 10^5^, red; 10^4^, blue; 10^3^, green; 10^2^, cyan; 10^1^, dark khaki; negative control, orange.
(DOCX)

S4 Fig DENV1-3 RT-RPA assay with 10^4^ analytical sensitivity. Fluorescence development via real-time detection by using a dilution range of 10^7^–10^1^ RNA molecules/μl of the DENV1-3 molecular standard. 10^7^ represented by black line; 10^6^, gray; 10^5^, red; 10^4^, blue; 10^3^, green; 10^2^, cyan; 10^1^, dark khaki; negative control, orange.
(DOCX)

S5 Fig RPA mobile laboratory in a local hospital in Bandafasi in Kedougou, Senegal. A, preparation to transfer the RPA mobile laboratory. B, Bandafasi in Kedougou region. C, local hospital. D, laboratory at the local hospital. E, Power supply from motor vehicle battery and convertor. F, RPA mobile laboratory operated by power from motor vehicle battery.
(DOCX)

S6 Fig Comparison between RT-RPA (Y-axis) and real-time RT-PCR (X-axis) for the detection of DENV in 90 clinical samples collected in Thailand between 2012–2013. Linear regression analysis of RT-RPA threshold time in minutes (TT, Y-axis) and real-time RT-PCR cycle threshold values (Ct, X-axis) were determined using PRISM. R squared value was 0.179.
(DOCX)

S7 Fig Alignment of DENV RT-RPA primers and exo-probe sequences with the consensus sequences of 3’NTR of DENV1-4 using Geneious (V: 6.1.5, Biomatters Limited, New Zealand). (DOCX)

S1 Table Primer and probe set sequences of the real-time RT-PCR performed during the field trial in Thailand. (DOCX)

S2 Table Results of screening spiked plasma samples with inactivated whole DENV1-4 with real-time RT-PCR and RT-RPA assays. (DOCX)

S3 Table Results of testing 90 DENV-positive RNA extracts using DENV RT-RPA assays and real-time RT-PCR. (DOCX)
