# Supplementary material for: Recombinase Polymerase Amplification Assay for Rapid Diagnostics of Dengue Infection
Source: PLoS One. 2015 Jun 15;10(6):e0129682. doi: 10.1371/journal.pone.0129682 (PMC4468249; doi:10.1371/journal.pone.0129682)
Supplement: S1 Fig — Three RPA exo probes (P), 19 forward primers (FP), and 5 reverse primers (RP) were tested to select combinations yielding the highest analytical DENV1-3 RT-RPA sensitivity. FP13, RP4, and P3 produced the best RT-RPA assay sensitivity. NNN are sites of the quencher and fluorophore in following order (BHQ1-dT) (Tetrahydrofuran) (FAM-dT). RC is the reverse complementary of the original sequence used in the experiment. (DOCX) [file pone.0129682.s001.docx]

--------+---------+---------+---------+---------+---------+---------+---------+---------+---------+---------+---------+---------+--------

110 120 130 140 150 160 170 180 190 200 210 220 230

--------+---------+---------+---------+---------+---------+---------+---------+---------+---------+---------+---------+---------+--------

DENV1-3 Amplicon AAGGACTAGAGGTTATAGGAGACCCCCCGCAAACAAAAACAGCATATTGACGCTGGGAGAGACCAGAGATCCTGCTGTCTCCTCAGCATCATTCCAGGCACAGAACGCCAGAAAATGGAATGGTGCTGTTGAATCAA

DENV1-3 RPA P1 ...........................................ATATTGACGCTGGG**NNN**GACCAGAGATCCTGCTGTCTCCTCAGCATCATTC...........................................

DENV1-3 RPA P2 ...............................................TGACGCTGGG**NNN**GACCAGAGATCCTGCTGTCTCCTCAGCATCATT............................................

DENV RPA P3 ...........................................ATATTGACGCTGGGAGAGACCAGAGATCCTGC**NNN**CTCCTCAGCATCATTC...........................................

DENV1-3 RPA FP1 ............................................................................GTCTCCTCAGCATCATTCCAGGCACAGAAC...............................

DENV1-3 RPA FP2 ...............................................................................TCCTCAGCATCATTCCAGGCACAGAAC...............................

DENV1-3 RPA FP3 .........................................................................GCTGTCTCCTCAGCATCATTCCAGGCACAGAAC...............................

DENV1-3 RPA FP4 ...............................................................................................AGGCACAGAACGCCAGAAAATGGAATGGTGCTGTT.......

DENV1-3 RPA FP5 ..........................................................................................ATTCCAGGCACAGAACGCCAGAAAATGGAAT................

DENV1-3 RPA FP6 ....................................................................................................CAGAACGCCAGAAAATGGAATGGTGCTGTTGA.....

DENV1-3 RPA FP7 ....................................................................................................CAGAACGCCAGAAAATGGAATGGTGCTGTTGAATCA.

DENV1-3 RPA FP8 ..............................................................................................CAGGCACAGAACGCCAGAAAATGGAATGGTGCTGT........

DENV1-3 RPA FP9 ...................................................................................................ACAGAACGCCAGAAAATGGAATGGTGCTGTTGAAT...

DENV1-3 RPA FP10 ..................................................................................................CACAGAACGCCAGAAAATGGAATGGTGCTGTTGAA....

DENV1-3 RPA FP11 ................................................................................................GGCACAGAACGCCAGAAAATGGAATGGTGCTGTTG......

DENV1-3 RPA FP12 .................................................................................................GCACAGAACGCCAGAAAATGGAATGGTGCTGTTGA.....

DENV1-3 RPA FP13 ..................................................................................................CACAGAACGCCAGAAAATGGAATGGTGCTGTTGAAT...

DENV1-3 RPA FP14 ...................................................................................................ACAGAACGCCAGAAAATGGAATGGTGCTGTTGAAT...

DENV1-3 RPA FP15 ..............................................................................................CAGGCACAGAACGCCAGAAAATGGAATGGTGCTGT........

DENV1-3 RPA FP16 .............................................................................................CCAGGCACAGAACGCCAGAAAATGGAATGGTGCTG.........

DENV1-3 RPA FP17 ............................................................................................TCCAGGCACAGAACGCCAGAAAATGGAATGGTGCT..........

DENV1-3 RPA FP18 ...........................................................................................TTCCAGGCACAGAACGCCAGAAAATGGAATGGTGC...........

DENV1-3 RPA FP19 ..........................................................................................ATTCCAGGCACAGAACGCCAGAAAATGGAATGGTG............

DENV1-3 RPA RP1 AAGGACTAGAGGTTAGAGGAGACCCCCC.............................................................................................................

DENV1-3 RPA RP2 AAGGACTAGAGGTTAGAGGAGACCCC...............................................................................................................

DENV1-3 RPA RP3 .AGGACTAGAGGTTAGAGGAGACC.................................................................................................................

DENV1-3 RPA RP4 .....................................AACAGCATATTGACGCTGGGAGAGACCAGAGATC..................................................................

DENV1-3 RPA RP5 .AGGACTAGAGGTTAGAGGAGAC..................................................................................................................

**S1 Fig. DENV1-3 RT-RPA primers and probes sequence aligned with the DENV1-3 amplicon.** Three RPA exo probes (P), 19 forward primers (FP), and 5 reverse primers (RP) were tested to select combinations yielding the highest analytical DENV1-3 RT-RPA sensitivity. FP13, RP4, and P3 produced the best RT-RPA assay sensitivity. NNN are sites of the quencher and fluorophore in following order (BHQ1-dT) (Tetrahydrofuran) (FAM-dT). RC is the reverse complementary of the original sequence used in the experiment.
