# Supplementary material for: Recombinase Polymerase Amplification Assay for Rapid Diagnostics of Dengue Infection
Source: PLoS One. 2015 Jun 15;10(6):e0129682. doi: 10.1371/journal.pone.0129682 (PMC4468249; doi:10.1371/journal.pone.0129682)
Supplement: S2 Fig — Two RPA exo probes (P), 3 forward primers (FP), and 2 reverse primers (RP) were tested to select combinations yielding the highest analytical DENV4 RT-RPA sensitivity. FP3, RP2, and P3 produced the best RT-RPA assay sensitivity. NNN are sites of the quencher and fluorophore in following order (BHQ1-dT) (Tetrahydrofuran) (FAM-dT). RC is the reverse complementary of the original sequence used in the experiment. (DOCX) [file pone.0129682.s002.docx]

---------+---------+---------+---------+---------+---------+---------+---------+---------+---------+---------+

10 20 30 40 50 60 70 80 90 100 110

---------+---------+---------+---------+---------+---------+---------+---------+---------+---------+---------+

Amplicon DEN4 CACAAAAACAGCATATTGACGCTGGGAAAGACCAGAGATCCTGCTGTCTCTGCAACATCAATCCAGGCACAGAGCGCCGCAAGATGGATTGGTGTTGTTGATCCAACAGG

DEN4 RPA P1 ............ATATTGACGCTGGGAAAGACCAGAGATCCTGC**NNN**CTCTGCAACATCAATC...............................................

DENV RPA P3 ............ATATTGACGCTGGGA**G**AGACCAGAGATCCTGC**NNN**CTC**CT**CA**G**CATCA**T**TC...............................................

DENV4 RPA FP1 ...................................................................CACAGAGCGCCGCAAGATGGATTGGTGTTGTTGAT........

DENV4 RPA FP2 .................................................................................AGATGGATTGGTGTTGTTGATCCAACAGG

DENV4 RPA FP3 .........................................................TCAATCCAGGCACAGAGCGCCGCAAGATG........................

DENV4 RPA RP1 ......AACAGCATATTGACGCTGGGAAAGACCAGAGATC......................................................................

DENV4 RPA RP2 CACAAAAACAGCATATTGACGCTGGGAAAG................................................................................

**S2 Fig. DENV4 RT-RPA primers and probes sequences aligned with the DENV4 amplicon.** Two RPA exo probes (P), 3 forward primers (FP), and 2 reverse primers (RP) were tested to select combinations yielding the highest analytical DENV4 RT-RPA sensitivity. FP3, RP2, and P3 produced the best RT-RPA assay sensitivity. NNN are sites of the quencher and fluorophore in following order (BHQ1-dT) (Tetrahydrofuran) (FAM-dT). RC is the reverse complementary of the original sequence used in the experiment.
