# Supplementary material for: Recombinase Polymerase Amplification Assay for Rapid Diagnostics of Dengue Infection
Source: PLoS One. 2015 Jun 15;10(6):e0129682. doi: 10.1371/journal.pone.0129682 (PMC4468249; doi:10.1371/journal.pone.0129682)
Supplement: S4 Fig — Fluorescence development via real-time detection by using a dilution range of 107–101 RNA molecules/μl of the DENV1-3 molecular standard. 107 represented by black line; 106, gray; 105, red; 104, blue; 103, green; 102, cyan; 101, dark khaki; negative control, orange. (DOCX) [file pone.0129682.s004.docx]

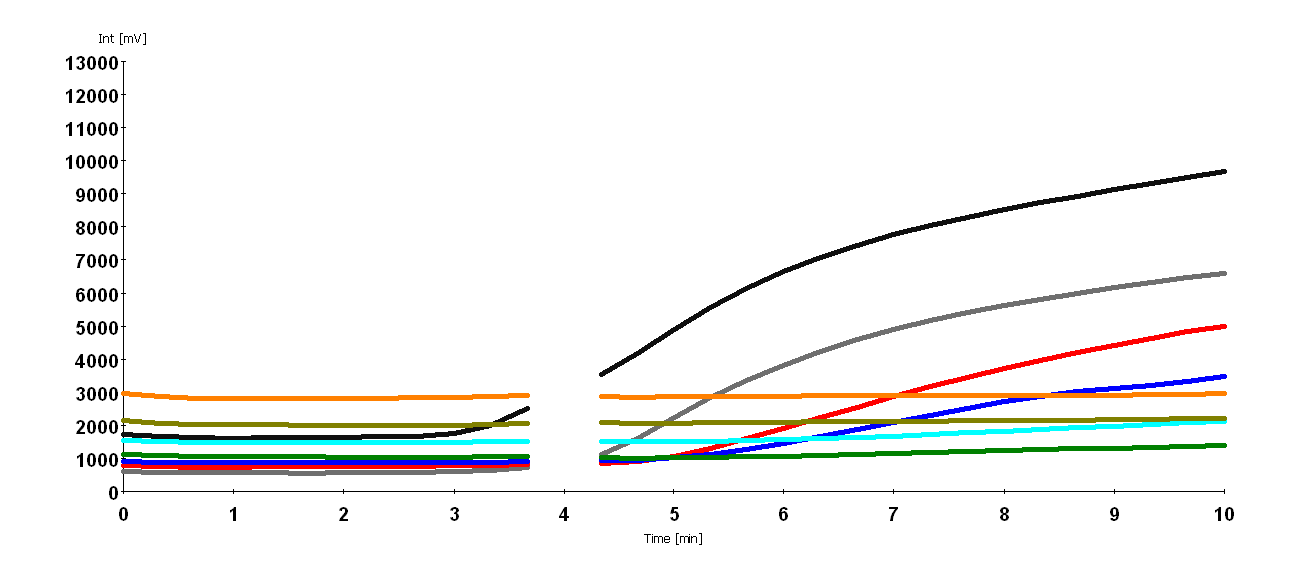


**S4 Fig. DENV1-3 RT-RPA assay with 10^4^ analytical sensitivity.** Fluorescence development via real-time detection by using a dilution range of 10^7^-10^1^ RNA molecules/µl of the DENV1-3 molecular standard. 10^7^ represented by black line; 10^6^, gray; 10^5^, red; 10^4^, blue; 10^3^, green; 10^2^, cyan; 10^1^, dark khaki; negative control, orange.
