# Supplementary material for: Recombinase Polymerase Amplification Assay for Rapid Diagnostics of Dengue Infection
Source: PLoS One. 2015 Jun 15;10(6):e0129682. doi: 10.1371/journal.pone.0129682 (PMC4468249; doi:10.1371/journal.pone.0129682)
Supplement: S5 Fig — A, preparation to transfer the RPA mobile laboratory. B, Bandafasi in Kedougou region. C, local hospital. D, laboratory at the local hospital. E, Power supply from motor vehicle battery and convertor. F, RPA mobile laboratory operated by power from motor vehicle battery. (DOCX) [file pone.0129682.s005.docx]

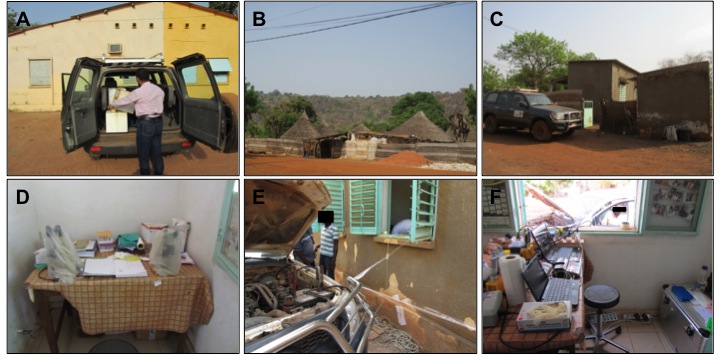


**S5 Fig. RPA mobile laboratory in a local hospital in Bandafasi in Kedougou, Senegal.** A, preparation to transfer the RPA mobile laboratory. B, Bandafasi in Kedougou region. C, local hospital. D, laboratory at the local hospital. E, Power supply from motor vehicle battery and convertor. F, RPA mobile laboratory operated by power from motor vehicle battery.
