# Supplementary material for: Recombinase Polymerase Amplification Assay for Rapid Diagnostics of Dengue Infection
Source: PLoS One. 2015 Jun 15;10(6):e0129682. doi: 10.1371/journal.pone.0129682 (PMC4468249; doi:10.1371/journal.pone.0129682)
Supplement: S6 Fig — Linear regression analysis of RT-RPA threshold time in minutes (TT, Y-axis) and real-time RT-PCR cycle threshold values (Ct, X-axis) were determined using PRISM. R squared value was 0.179. (DOCX) [file pone.0129682.s006.docx]

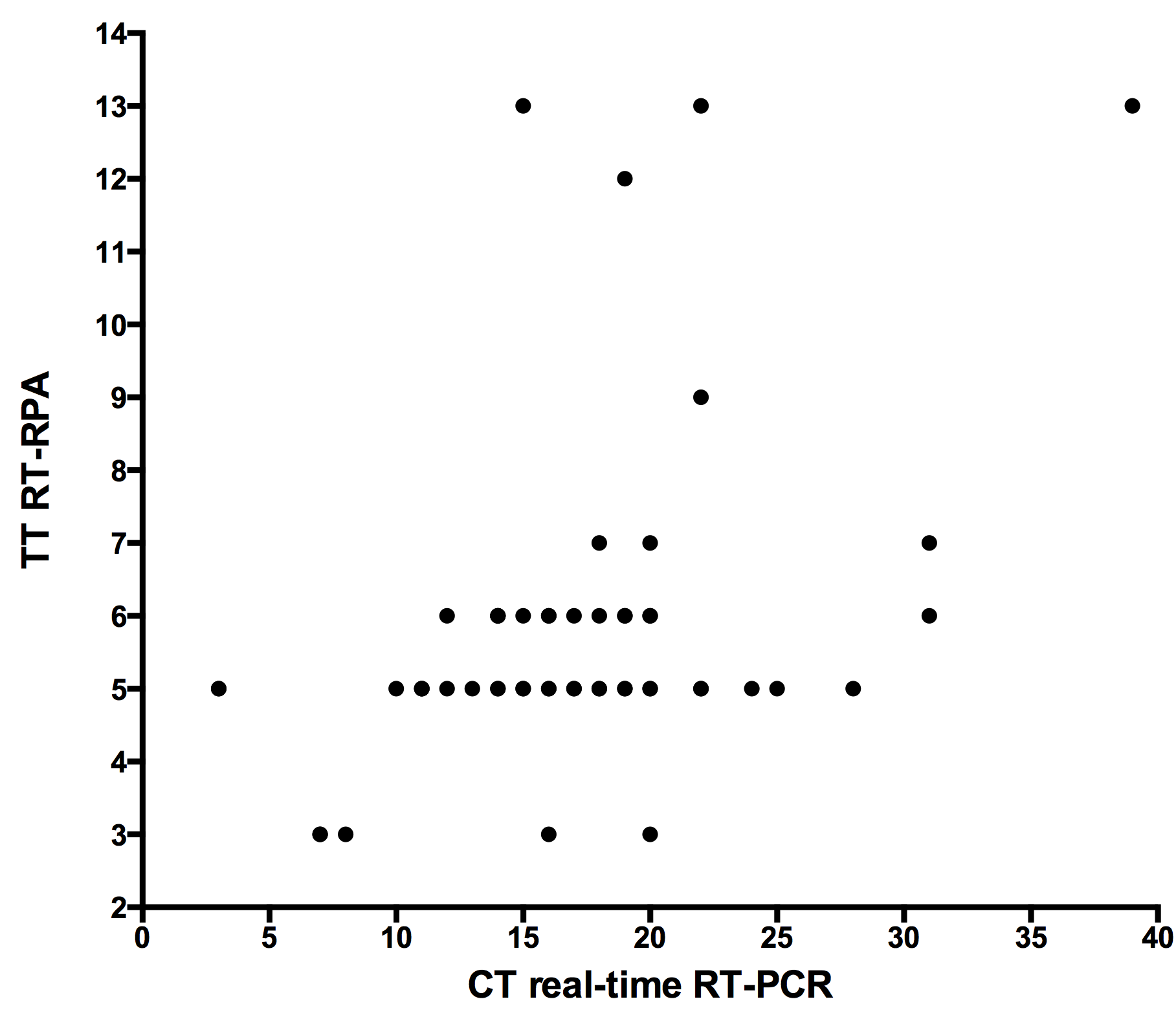


**S6 Fig. Comparison between RT-RPA (Y-axis) and real-time RT-PCR (X-axis) for the detection of DENV in 90 clinical samples collected in Thailand between 2012-2013.** Linear regression analysis of RT-RPA threshold time in minutes (TT, Y-axis) and real-time RT-PCR cycle threshold values (Ct, X-axis) were determined using PRISM. R squared value was 0.179.
