# Supplementary material for: Recombinase Polymerase Amplification Assay for Rapid Diagnostics of Dengue Infection
Source: PLoS One. 2015 Jun 15;10(6):e0129682. doi: 10.1371/journal.pone.0129682 (PMC4468249; doi:10.1371/journal.pone.0129682)
Supplement: S7 Fig — (DOCX) [file pone.0129682.s007.docx]

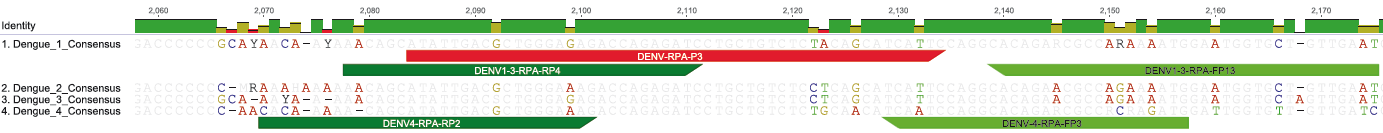


**S7 Fig. Alignment of DENV RT-RPA primers and exo-probe sequences with the consensus sequences of 3´NTR of DENV1-4 using Geneious (V: 6.1.5, Biomatters Limited, New Zealand).**
