# Supplementary material for: Recombinase Polymerase Amplification Assay for Rapid Diagnostics of Dengue Infection
Source: PLoS One. 2015 Jun 15;10(6):e0129682. doi: 10.1371/journal.pone.0129682 (PMC4468249; doi:10.1371/journal.pone.0129682)
Supplement: S1 Table — (DOCX) [file pone.0129682.s008.docx]

**S1 Table. Primer and probe set sequences of the real-time RT-PCR performed during the field trial in Thailand.**

| **Serotype** | | **Primer sequence (5’-3’)** | **Position** | **Product size**  **in base** |
| --- | --- | --- | --- | --- |
| DEN1-Forward | | ATCCATGCCCATCACCAAT | 9865-9883 | 100 |
| DEN1-Reverse | | TGTGGGTTTTGTCCTCCATC | 9945-9964 |  |
| DEN2-Forward | | TCCATACACGCCAAACATGAA | 9859-9879 | 125 |
| DEN2-Reverse | | GGGATTTCCTCCCATGATTCC | 9963-9983 |  |
| DEN3-Forward | | TTTCTGCTCCCACCACTTTC | 9591-9610 | 118 |
| DEN3-Reverse | | CCATCCYGCTCCTTGAGA | 9691-9708 |  |
| DEN4-Forward | | GYGTGGTGAAGCCYCTRGAT | 9587-9607 | 178 |
| DEN4-Reverse | | AGTGARCGGCCATCCTTCAT | 9744-9764 |  |
| Probe | Probe sequences | | | |
| DEN1-probe | 5’(FAM) TCAGTGTGGAATAGGGTTTGGATAGAGGAA 3’(BHQ-1) | | | |
| DEN2-probe | 5’(FAM) AGGGTGTGGATTCGAGAAAACCCATGG 3’(BHQ-1) | | | |
| DEN3-probe | 5’(Texas Red) AAGAAAGTTGGTAGTTCCCTGCAGACCCCA 3’(BHQ-2) | | | |
| DEN4-probe | 5’(Texas Red) ACTTCCCTCCTCTTYTTGAACGACATGGGA 3’(BHQ-2) | | | |
