# Supplementary material for: Recombinase Polymerase Amplification Assay for Rapid Diagnostics of Dengue Infection
Source: PLoS One. 2015 Jun 15;10(6):e0129682. doi: 10.1371/journal.pone.0129682 (PMC4468249; doi:10.1371/journal.pone.0129682)
Supplement: S2 Table — (DOCX) [file pone.0129682.s009.docx]

**S2 Table. Results of screening spiked plasma samples with inactivated whole DENV1-4 with real-time RT-PCR and RT-RPA assays.**

| **Sample name** | **Real-time RT-PCR** | | **DENV1-3 RT-RPA** | **DENV4 RT-RPA** |
| --- | --- | --- | --- | --- |
|  |  |  |  |  |
|  | **Ct Value** | **Genome copies/rxn** | **TT Value** | **TT Value** |
| DEN1 | 27,25 | 1,50E+04 | 4,7 | neg |
| DEN | 30,52 | 1,63E+03 | 5,7 | neg |
| DEN1 | 33,37 | 2,37E+02 | 6,3 | neg |
| DEN2 | 26,09 | 3,28E+04 | 4,3 | 5,3 |
| DEN2 | 28,75 | 5,43E+03 | 5,3 | neg |
| DEN2 | 31,95 | 6,18E+02 | 6,3 | neg |
| DEN3 | 26,51 | 2,47E+04 | 5 | neg |
| DEN3 | 29,29 | 3,76E+03 | 5,3 | neg |
| DEN3 | 32,74 | 3,63E+02 | 6,3 | neg |
| DEN4 | 25,98 | 3,55E+04 | neg | 3,7 |
| DEN4 | 29,51 | 3,23E+03 | neg | 5,3 |
| DEN4 | 32,66 | 3,83E+02 | neg | 5,7 |
| Nc | No Ct | NA | neg | neg |

Nc, negative control; neg, negative; NA, non-applicable; TT, Threshold time
