# Supplementary material for: Recombinase Polymerase Amplification Assay for Rapid Diagnostics of Dengue Infection
Source: PLoS One. 2015 Jun 15;10(6):e0129682. doi: 10.1371/journal.pone.0129682 (PMC4468249; doi:10.1371/journal.pone.0129682)
Supplement: S3 Table — (DOCX) [file pone.0129682.s010.docx]

**S3 Table. Results of testing 90 DENV-positive RNA extracts using DENV RT-RPA assays and real-time RT-PCR.**

| **Sample number** | **RT-RPA** | **Real-time RT-PCR** |
| --- | --- | --- |
|  | **minutes** | **Ct** |
| **DEN-1** | | |
| 1 | 13.3 | 39 |
| 2 | 5.3 | 20 |
| 3 | neg | 30 |
| 4 | 6 | 16 |
| 5 | neg | 30 |
| 6 | 6 | 14 |
| 7 | neg | 34 |
| 8 | 5.3 | 28 |
| 9 | 5.3 | 11 |
| 10 | 5.3 | 16 |
| 11 | 5.3 | 20 |
| 12 | 3.7 | 20 |
| 13 | neg | 14 |
| 14 | 3.3 | 16.01 |
| 15 | 5.3 | 19.59 |
| 16 | 5.3 | 19.41 |
| 17 | neg | 23.06 |
| 18 | 5.7 | 18.61 |
| 19 | 5.7 | 15.42 |
| 20 | neg | 27.67 |
| 21 | 6 | 18.61 |
| 22 | neg | 20.31 |
| 23 | 6 | 15.33 |
| 24 | neg | 34.64 |
| **DEN-2** | | |
| 25 | neg | 25.58 |
| 26 | 5.7 | 13.89 |
| 27 | neg | 20.76 |
| 28 | neg | 21.12 |
| 29 | 5.7 | 17.23 |
| 30 | 6 | 20.38 |
| 31 | 6 | 15.79 |
| 32 | neg | 25.8 |
| 33 | neg | 32 |
| **DEN-3** | | |
| 34 | 3.3 | 7.7 |
| 35 | 3 | 8.75 |
| 36 | 6 | 14.45 |
| 37 | neg | 20.29 |
| 38 | 13.3 | 41 |
| 39 | 12.3 | 19 |
| 40 | 13.7 | 22 |
| 41 | neg | 16 |
| 42 | 9 | 22 |
| 43 | 5.3 | 15 |
| 44 | 5.7 | 25 |
| 45 | 5.3 | 16.3 |
| 46 | 5.3 | 14 |
| 47 | 5.3 | 24 |
| 48 | neg | 27 |
| 49 | neg | 27 |
| 50 | 13.3 | 15 |
| 51 | 6 | 31 |
| 52 | 13.7 | 34 |
| 53 | 3 | 7.68 |
| 54 | 5.7 | 10.97 |
| 55 | 5.3 | 11.09 |
| 56 | 5.3 | 18.03 |
| 57 | neg | 24.58 |
| 58 | 5.7 | 21.9 |
| 59 | 5.7 | 16.04 |
| 60 | 5.7 | 3.25 |
| 61 | 5.7 | 22.69 |
| 62 | 6.3 | 20.85 |
| 63 | 6.3 | 19.34 |
| 64 | 6 | 12.77 |
| 65 | neg | 24.91 |
| 66 | 6 | 16.04 |
| 67 | 5.7 | 12.67 |
| 68 | 5.7 | 11.76 |
| 69 | neg | 23.02 |
| 70 | 5.7 | 3.56 |
| 71 | 5.7 | 22.96 |
| 72 | neg | 22.3 |
| 73 | Very weak signal | 16.39 |
| 74 | 5.3 | 14.13 |
| 75 | 6 | 14.95 |
| **DEN-4** | | |
| 76 | neg | 29.21 |
| 77 | neg | 22.39 |
| 78 | 7 | 18.69 |
| 79 | 6 | 17.62 |
| 80 | 7 | 31.32 |
| 81 | 5.3 | 17.16 |
| 82 | 7 | 20.49 |
| 83 | 6 | 19.11 |
| 84 | neg | 24.62 |
| 85 | neg | 19.11 |
| **DENV serotype was not identified** | | |
| 86 | 5.7 | neg |
| 87 | 5.7 | neg |
| 88 | 6 | neg |
| 89 | 13.3 | neg |
| 90 | 5.3 | neg |
